# Inversion of inherited thrusts by wastewater injection induced seismicity at the Val d’Agri oilfield (Italy)

**DOI:** 10.1038/srep37165

**Published:** 2016-11-14

**Authors:** M. Buttinelli, L. Improta, S. Bagh, C. Chiarabba

**Affiliations:** 1 Istituto Nazionale di Geofisica e Vulcanologia, Department of Seismology and Tectonophysics, Rome, Italy, Via di Vigna Murata 605, 00143, Roma, Italy; 2 Istituto Nazionale di Geofisica e Vulcanologia, National Earthquake Centre, Rome, Italy

## Abstract

Since 2006 wastewater has been injected below the Val d’Agri Quaternary basin, the largest on-land oilfield in Europe, inducing micro-seismicity in the proximity of a high-rate injection well. In this study, we have the rare opportunity to revise a massive set of 2D/3D seismic and deep borehole data in order to investigate the relationship between the active faults that bound the basin and the induced earthquakes. Below the injection site we identify a Pliocene thrusts and back-thrusts system inherited by the Apennines compression, with no relation with faults bounding the basin. The induced seismicity is mostly confined within the injection reservoir, and aligns coherently with a NE-dipping back-thrust favorably oriented within the current extensional stress field. Earthquakes spread upwards from the back-thrust deep portion activating a 2.5-km wide patch. Focal mechanisms show a predominant extensional kinematic testifying to an on-going inversion of the back-thrust, while a minor strike-slip compound suggests a control exerted by a high angle inherited transverse fault developed within the compressional system, possibly at the intersection between the two fault sets. We stress that where wastewater injection is active, understanding the complex interaction between injection-linked seismicity and pre-existing faults is a strong requisite for safe oilfield exploitation.

Induced seismicity is attracting the attention of the scientific community, especially after moderate-to-large earthquakes caused by the injection of wastewater from hydrocarbon exploitation into deep wells (e.g. the M_w_ 5.7 Prague, Oklahoma and M_w_ 5.3 Raton basin, Colorado sequences^1^,^2^). Long-term water disposal by high-rate injection wells can induce small-to-moderate earthquakes, remobilizing pre-existing faults as a consequence of pore pressure increase^3^,^4^,^5^,^6^,^7^. The lack of knowledge on the presence and geometry of even small fault segments at depth may result in misquoting the anthropogenic hazard and cause undesired scenarios and economic loss^8^,^9^,^10^.

The Val d’Agri (VDA) hosts a giant oilfield, the largest on land in Europe, and additionally it is one of the areas of highest seismic hazard in Italy. Since the 1990 s the VDA oilfield produces hydrocarbons from a high-productive, fractured limestones reservoir. The oil production rate is around 90 k bbl/day. Starting on June 2006, the water associated to hydrocarbons exploitation is mostly re-injected into an unproductive marginal portion of the reservoir (Fig. 1) by a high-rate injection well (Costa Molina 2, CM2). A spatio-temporal relationship between micro-seismicity (M_L_ ≤ 2.2) and wastewater injection has been observed^11^,^12^, with hypocenters that align along a NE-dipping structure located a few kilometers to the SW of the well. The aim of this work is to understand the potential of earthquakes in the area by investigating the presence of faults near the injection well, their extent, geometry and relation with the major Quaternary faults that bound the extensional basin. We analyzed a large dataset of 2D/3D seismic profiles and deep boreholes, in order to image the crustal structure in the volume around the injection well and to compare the retrieved faults with precisely located hypocenters of induced earthquakes.

## Geologic and seismotectonic outline

**Figure 1:**
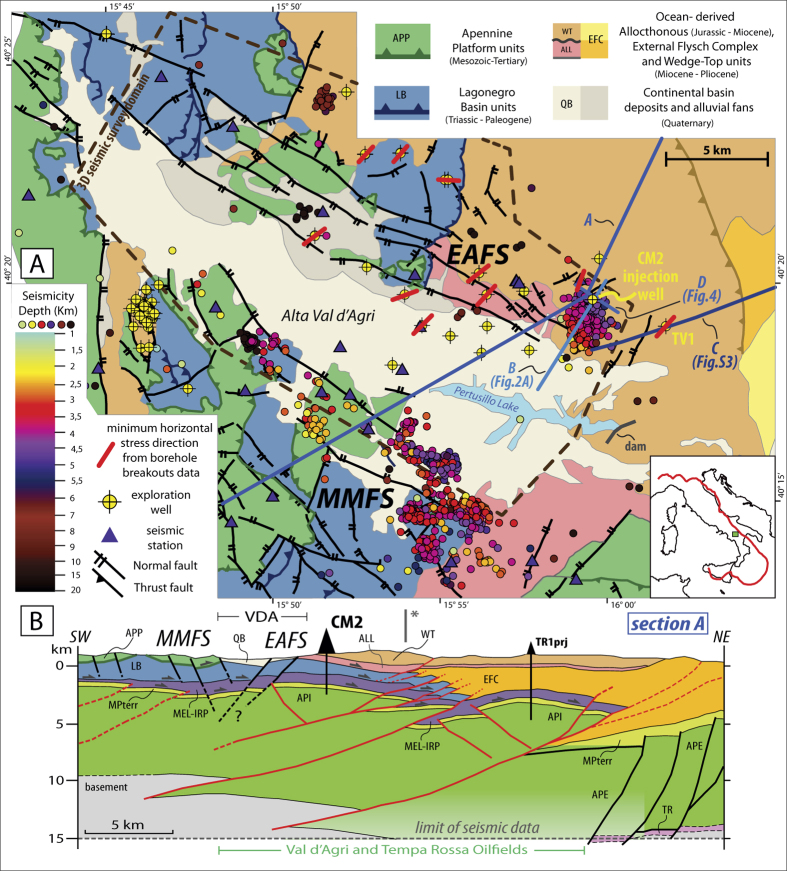
(**A**) Geological map of the VDA region (redrawn after ref. 20) reporting: seismic stations (triangles), the 2001–2014 seismicity as a function of hypocentral depth (coloured circles), exploration wells (yellow circles). The red lines on wells indicate minimum horizontal stress directions inferred from borehole breakouts. The EAFS and MMFS fault systems are redrawn after refs 15 and 19. Blue lines report the traces of 4 sections discussed in the manuscript. (**B**) Schematic geological section across the VDA illustrating the axial and external sectors of the southern Apennines (modified after ref. 10). TR-Permo-Triassic clastic sequences. APE-External Apulia Platform (Mesozoic-Tertiary), API-Internal Apulia Platform (Mesozoic-Tertiary); MPterr-Late Miocene-Lower Pliocene terrigenous unconformable deposits covering the API; MEL-IRP: mèlange layer (Late Miocene-Lower Pliocene) and undifferentiated Miocene Flysch; LB-Lagonegro basin units (Mesozoic- Paleogene); APP-internal Apennine Platform (Mesozoic-Tertiary); EFC-marly-calcareous sequences and Miocene flysch deposits (External Flysch Complex); ALL-WT-allochthonous units of the Internal Apenninic Nappe (Albidona Formation, Eocene-Miocene) and related wedge-top deposits (Gorgoglione Formation, Middle-Upper Miocene); QB-VDA Quaternary basin; CM2-Costa Molina 2 well; TR1-Tempa Rossa 1 well; TV1 – Tempa del Vento 1 well. The I* symbol marks the end of the section trace A reported in map, while the geological section extends further northeastward to illustrate the structure of the external portion of the belt. [this figure has been constructed using Esri ArcGIS ArcMap 10.2.1 http://www.esri.com/software/arcgis/arcgis-for-desktop for Desktop and Move2016.1 http://www.mve.com/software/move].

The VDA is a Quaternary basin developed within the southern Apennines thrust-and-fold belt (Fig. 1A), when an extensional phase replaced a Mio-Pliocene compression^13^ among others. The evolution of the compressional belt consisted of two distinct phases with different tectonic style (Fig. 1B): i) a Neogene thin-skinned phase, where a pile of thrust sheets consisting of Meso-Cenozoic sedimentary units (internal Apennine Platform and Lagonegro Basin) with associated foredeep flysch deposits stacked over the Inner Apulian Platform (API); ii) a subsequent Late Pliocene-Early Pleistocene thick-skinned phase during which high angle N- to NW-trending thrusts (with antithetic back thrusts) were deeply rooted originating large-wavelength anticlines within the API^14^,^15^,^16^. The API consists of a 6–8 km thick succession of Meso-Cenozoic shallow-water carbonates, and the carbonate anticlines form the oilfield main traps, with Pliocene siliciclastic foredeep deposits acting as the seal for the accumulation of hydrocarbons. The buried API is separated from the upper allocthonous units by a Pliocene mélange layer which has a variable thickness locally reaching ∼1500 m which corresponds to a regional detachment level (Fig. 1B).

Since the Middle Pleistocene, the area underwent a NE-trending extension, where deformation rate was about 2–5 mm/yr^17^. The VDA extensional basin is shaped by two active and opposite dipping, high-angle range-bounding fault systems: the Eastern Agri (EAFS) and the Monti della Maddalena (MMFS) (Fig. 1A)^18^,^19^. Both the EAFS and MMFS consist of arrays of separated normal fault segments, a typical feature of a set of immature fault systems. Large normal-faulting earthquakes occurred along the axial sector of the southern Apennines^20^ and struck the VDA area, the last of which is the 1857 M7 event. Although the causative fault is still debated, recent studies associated the 1857 earthquake with one of the MMFS segments^21^.

Instrumental seismicity catalogues reported only rare and sparse low-magnitude earthquakes (Fig. 1A). High-resolution studies of background seismicity evidenced that the shallow seismicity was spatially correlated to the southernmost fault segments of the MMFS (Fig. 1A). Low-magnitude seismicity was related to the seasonal variations of the Pertusillo water impoundment located a few kilometers to the South of the oilfield^22^,^23^ (Fig. 1A).

## 3D structural model of the injection area

We build a 3D structural model of the crustal volume around the CM2 well, by analyzing a large amount of hydrocarbon exploration data that includes: *i*) time-migrated reflection data from a 3D survey; *ii*) stack and time-migrated reflection profiles *iii*) geophysical and stratigraphic logs from more than 20 deep wells (Fig. S2). The quality of seismic reflection data within the VDA is variable and strongly influenced by near-surface geology and topographic conditions^17^. The seismic response is generally poor under the Quaternary basin due to the presence of thick, unconsolidated continental clastic deposits. Conversely, seismic imaging is fair to good in the eastern and northeastern sectors (the latter is present in the injection area), where Tertiary marly-clayey and flysch deposits outcrop (Fig. 1A). Due to the extreme structural complexity, the use of a large deal of calibration wells is of key importance for a reliable seismic interpretation and 3D model construction (Fig. S2).

**Figure 2:**
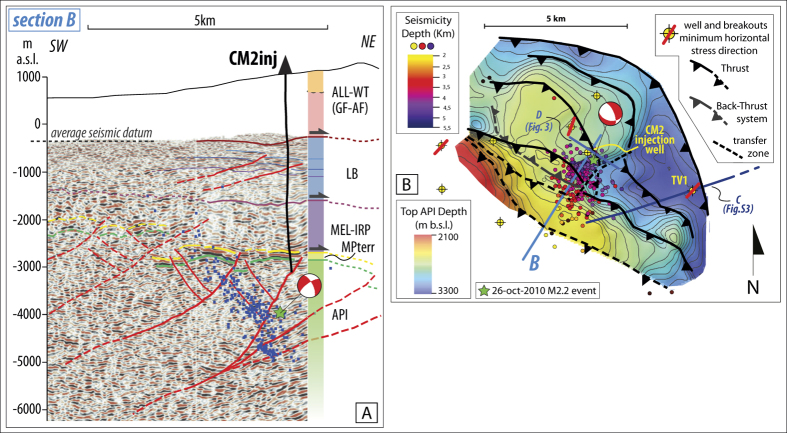
(**A**) Interpretation of the depth converted vertical slice extracted from the 3D seismic volume tied to the CM2 well. The section trace is showed in Fig. 1A (section B). The blue dots depict hypocenters of the 2006–2014 seismicity occurred close to the CM2 well. Main seismographic units codes references in Fig. 1; (**B**) Structural map of the top of API in the injection area and distribution of the 2006–2014 induced seismicity (In both panels the green star depicts the M_L_ 2.2 event accompanied by the focal mechanism). The map shows the main SW-dipping reverse faults, the sub-vertical transverse fault (dashed line), the NE-dipping back-thrust related to the induced seismicity and deep wells with associated borehole breakouts. Section traces in blue color. [this figure has been constructed using Move2016.1 http://www.mve.com/software/move and Esri ArcGIS ArcMap 10.2.1 http://www.esri.com/software/arcgis/arcgis-for-desktop].

The subsurface architecture of the API is dominated by upheaved long-wave anticline deforming the carbonate reservoir, un-harmonically covered by the stack of allochthonous units that progressively thicken eastwards (Figs 2A and S3, S4). According to previous studies^14^,^15^,^16^,^24^, thrust faults affecting the allochtonous stack sole on the mélange basal detachment and are not connected with the high angle reverse faults developed within the API, which in turn rarely propagate upwards (Figs 2A and S3, S4). The upper allochtonous units are characterized by well-organized high frequency reflectors defining a syncline filled by Miocene turbiditic terrigenous sequences, which are un-conformably covered by wedge-top deposits (Fig. S3). These siliciclastic sequences cover the Lagonegro Basin units and extensively outcrop to the East of the basin and in the injection area (Fig. 1), with a thickness that can exceed 1500 m.

For the depth conversion, we used a velocity model defined by combining 3D seismic check-shots data, Vertical Seismic Profiles (VSP) and sonic logs (Table S1), as well as other velocity models available in literature for the same areas of the southern Apennines^16^. The 3D depth converted data provide fair to good structural images of the thrust-and-fold system in the injection area (Figs 2A and S4). The antiform drilled by CM2 well is structured by at least four NW-trending, SW-dipping moderate-high angle reverse faults with associated NE-dipping back-thrusts. The thrusts and back-thrust are blind structures apparently ending up into an overlying mélange layer that mostly consists of ductile mudstones. Seismic data do not provide evidence for normal faults close to the injection well, within the carbonate reservoir or the allocthonous units (Figs 2A and S4).

**Figure 3:**
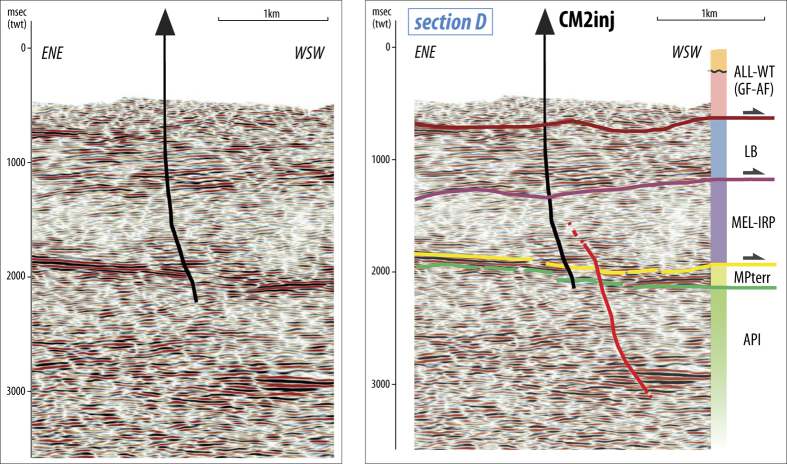
Un-interpreted and interpreted time-migrated vertical section (section trace D on Figs 1A and 2B) extracted from the 3-D survey. The section highlights a NNE-SSW trending sub-vertical transverse fault cutting the Apulian units close to the CM2 injection well. [this figure has been constructed using Move2016.1 http://www.mve.com/software/move].

The top API structural map (Fig. 2B) shows that the carbonate reservoir is shallower to the west (<2500 m) and deepens to the east (>3000 m). In addition, the API is affected by a NNE-trending step located to the southeast of the CM2 well. Such step corresponds to a sub-vertical transverse fault that cuts the reservoir and the mélange layer, slightly dipping to SE with a minor dextral strike-slip kinematic (Figs 2B and 3). The northwestern elevated sector coincides with the hanging wall blocks of two arcuate thrusts, whose lateral ramps die in the transverse step fault. The CM2 well is located in the hanging-wall block of the southern arcuate thrust. Disarticulation and complexity of the top reservoir indicate that transfer faults and arcuate thrusts developed to accommodate differential compression in adjacent portions of API.

## Fault systems and earthquake distribution

The retrieved faults were compared with accurate 3D locations of injection-linked seismicity recorded during the entire period of active injection. We integrated earthquake data recorded between 2001 and 2014 by a monitoring network managed by the local oilfield operator supplemented by INGV permanent and temporary stations installed in the VDA.

After re-picking of P and S phases, 3-D hypocentral locations were determined by a local earthquake tomography with an unprecedented spatial resolution for the region^25^ (see the electronic supplement for further details). A total of 248 events with 0.2 ≤ M_L_ ≤ 2.2 with a horizontal and vertical standard errors lower than 150 and 200 m were located within 5 km of well CM2 (Figs 1 and S1).

Events cluster between 2.5–5 km depth and concentrate within the API units, elongating in the SSW-NNE direction and defining a ∼50° NE-dipping fault with a down-dip extension of ∼4 km and a width of ∼2.5 km. Only a few, sparse events fall within the overlying, ductile mélange layer (Figs 2A,B and S4).

Focal mechanisms show predominant normal faulting kinematic striking from WNW-ESE to NW-SE, with the NE-dipping nodal plane coherent with the seismicity alignment at depth (Fig. S1). The induced earthquakes are located very close to one of the interpreted back-thrusts (Figs 2A and S4). Deeper events, including the M_L_ 2.2 largest earthquake, possibly relate to the more external reverse faults, whereas only a few earthquakes fall in the footwall block of the whole thrust system. The SSW-NNE elongated earthquakes cloud matches the right lateral ramps of the arcuate thrusts and back-thrusts developed in the northwestern upheaved sector of the reservoir (Fig. 2B). In addition, event distribution follows the trend of the sub-vertical transverse fault recognized in the API (Fig. 2B).

## Discussion

The set of faults present within the API carbonate reservoir close to the injection site consists of a system of NW-trending, SW-dipping arcuate thrusts and associated back-thrusts (Figs 2A,B and S4) that developed during the Late Pliocene-Early Pleistocene thick-skinned compressional phase. The entire system is elongated consistently with the Apenninic strike for about 8–10 km, but is segmented by a transverse high-angle fault (Figs 2B and 3). A set of blind, NE-dipping back-thrusts sole on two SW-dipping main thrusts. The seismic reflection data do not show any continuation of such thrusts and back-thrust toward the surface beyond the tectonic mélange layer covering the API. Moreover, there is no evidence of extensional faults to the SW of the CM2 injection well, indicating that the entire system of compressional structures is located in the footwall of the Quaternary normal fault-system bounding the VDA basin to the northeast (e.g. EAFS, Fig. 1A).

The induced earthquakes mostly align on one of the back-thrusts, having predominant normal fault focal mechanisms solutions. This result contrasts with previous interpretations that ascribed to an unknown normal-fault of the basin bounding system the observed microseismicity^11^. Such focal mechanisms are also coherent with the current NE-trending extensional local stress field inferred by borehole breakouts (Figs 1A and 2B) and S-wave anisotropy^12^,^26^,^27^. Taken together, these results provide compelling evidence of an ongoing inversion of inherited reverse faults in extensional regime.

**Figure 4:**
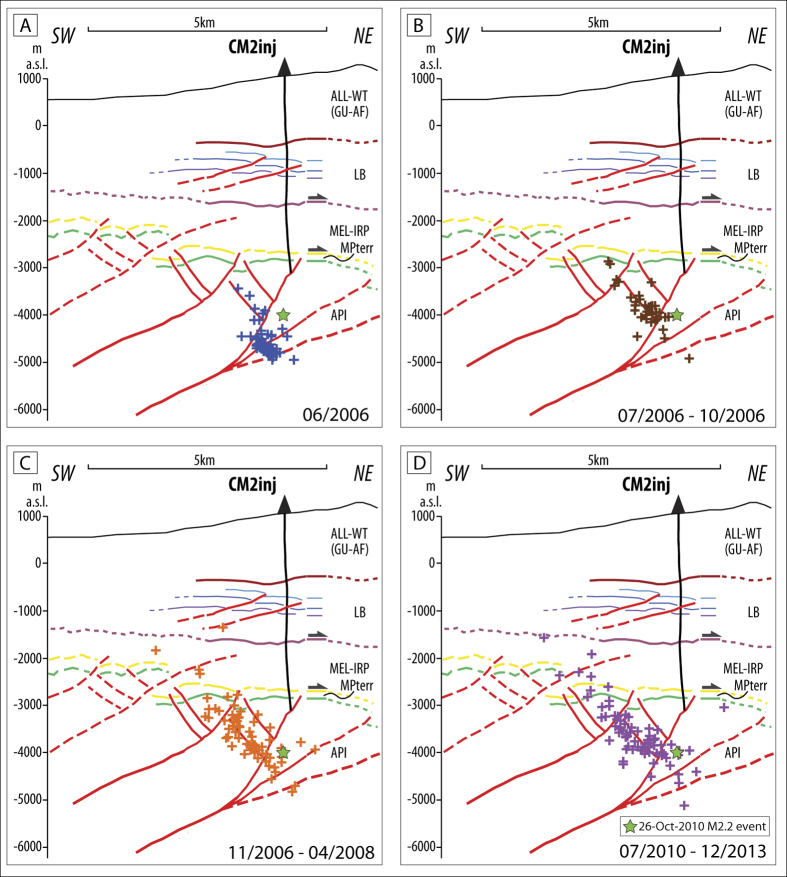
Spatio-temporal evolution of the 2006–2014 induced seismicity and its relationship with geological structures. The events (crosses) are reported for different time intervals to evidence seismicity migration. The largest M_L_ 2.2 earthquake is depicted in all panels by a green star. [this figure has been constructed using Move2016.1 http://www.mve.com/software/move].

During the early stages of injection (June-October 2006), seismicity migrated very quickly upwards from the deeper part of the reservoir along the back-thrust, for a length of about 2 km (Fig. 4A). The migration of seismicity continued with slower speed until April 2008, activating the entire vertical extent of the back-thrust with hypocenters confined within the carbonate reservoir (Fig. 4B,C). Further upward migration of seismicity was probably hampered by the rheological properties of the ductile mélange layer (Fig. 4D). The downward migration of seismicity could have been hindered by either the rooting of the back-thrust onto the SW-dipping main thrust that generated isolated fluid compartments, or the exceptionally low permeability of the API lower sequence consisting of Triassic evaporitic dolostones and anhydrites that hinders effective transmission of pore-pressure perturbations at depth (k = 10^−21^–10^−18^ m^2^)^28^, summed to the progressive increment of the confining pressure that tends to close fractures.

The fracture permeability inferred by hydraulic diffusivity is very high, in the order of 10^−13^ m^2 ^12, in agreement with the petro-physical model of the reservoir^29^,^30^. This suggests that in the carbonate reservoir pore-pressure diffusion and fluid migration from the 4 km deep well is controlled by a widespread system of open and conductive fractures, which helped a rapid propagation of pore pressure perturbations and the re-activation of the back-thrust, at least in the portion that is favorably oriented with the current extensional stress field. In this view, the back-thrust acted as preferential pathway for the propagation of pore-pressure perturbations.

Focal mechanisms strongly support the hypothesis of the control exerted by the inherited compressive structures on the injection-linked seismicity. The normal faulting solutions with predominant WNW-ESE to NW-SE striking nodal planes (Fig. S1) are consistent with the trend of reverse faults (Figs 2A,B and S4), while the few events with strike-slip high-angle kinematics might be related to the NNE-trending sub-vertical transverse fault (Figs 2B and 3). This finding suggests that the transverse fault plays a role on the occurrence of the induced seismicity concurrently with the NW-SE trending set of reverse faults.

It has been observed that fault zones can channel fluid flow and transmit injection-induced pore pressure even over large distances from injection areas^1^,^31^. Such pressure channeling effect has been related to the occurrence of induced earthquakes at the intersections with distant transverse faults that may act as flow barriers, trapping the pressure front within its damage zone and causing a more rapid pressure increase right at the junction between the two faults^32^.

For the VDA case study, the complex interaction between regional stress and pre-existing faults led to the segmentation of the fault system, limiting the lateral extent of faults that underwent failure due to pore pressure increase within the reservoir. The role of the NNE-trending transverse fault acting as a pore pressure flow barrier might be invoked to justify the peculiar arrangement of observed induced seismicity which is elongated coherently. In this view, a pressure channeling effect would occur at the intersection between the NW-trending back-thrusts and the NNE-trending high-angle transverse fault.

Although we do not find a direct relationship between induced seismicity and the Quaternary extensional faults bounding the basin, our results stimulate the discussion on whether critically stressed faults could be mobilized during long-term wastewater injection.

## Conclusions

By combining high-quality structural images of the VDA hydrocarbon reservoir defined by interpreting 2D/3D seismic and well data with high-accuracy hypocentral locations of microseismicity induced by injection well CM2, we provide compelling evidence of a strong control exerted by inherited geologic structures on the phenomena observed since 2006. Induced events with predominant normal-fault mechanisms concentrate along a blind back-thrust of a NW-trending, SW-dipping thrust-system developed during Pliocene in the Apulian carbonate reservoir that have no relation with Quaternary normal faults bounding the basin. Also, inherited structures controlled the spatio-temporal distribution of the seismicity that migrated up-dip along the back-thrust and remained almost confined within the deformed Apulian units. Re-activation of the back-thrust is due to the increase of pore-pressure in the water-saturated reservoir and was favored by its optimal orientation within the SW-NE oriented current extensional stress field. A transverse fault cuts the thrust-system with predominant lateral movement and likely contributes to the propagation of pore-pressure perturbations along a preferential pathway, possibly at the intersection between the two fault sets. The primary role of injection pressure on the observed micro-seismicity, with major swarms occurred during injection stages characterized by rapidly increasing pressure and/or pressure above 13–14 MPa has been already reported^12^. This key finding, combined with the evidence of a strict relation with a fault provided by our study, has implications not only for a deeper understanding of the mechanism for inducing seismicity, but also for managing disposal activity. For future injection operations, abrupt pressure variations should be avoided and the pressure should be maintained under the threshold of 13–14 MPa. Even if the seismicity rate has strongly declined over the last three years^33^, the future monitoring should include high-accuracy 3D hypocentral determinations and a careful analysis of the spatio-temporal evolution of seismicity in relation to the specific structural model presented in this study. In case of future swarms, this advanced monitoring strategy should be mandatory for a safe management of the injection operations because it could unravel changes in seismicity and possible migration along other well-oriented reservoir faults, as well as towards deeper basement faults. Lastly, our structural model could be used as reference to perform a geologically realistic 3D geomechanical modeling of the stress changes resulting from the combined effects of wastewater injection and hydrocarbon withdrawal from the reservoir. Such a modeling would enable to model the observed induced seismicity and to quantitatively assess the likelihood to trigger earthquakes on optimally oriented faults evidenced in our study^34^. At a more general scale, our results reinforce the need of a multidisciplinary approach combining advanced seismological analysis with reliable structural models of hydrocarbon reservoirs for a thorough understanding of induced seismicity. Such an integrated approach is fundamental in seismically active areas to discriminate natural and induced events and to unravel potential migration of seismicity towards large seismogenic faults.

## Additional Information

**How to cite this article**: Buttinelli, M. *et al*. Inversion of inherited thrusts by wastewater injection induced seismicity at the Val d’Agri oilfield (Italy). *Sci. Rep*. **6**, 37165; doi: 10.1038/srep37165 (2016).

**Publisher’s note**: Springer Nature remains neutral with regard to jurisdictional claims in published maps and institutional affiliations.

## Supplementary Material

Supplementary Information
