# Supplementary material for: Inversion of inherited thrusts by wastewater injection induced seismicity at the Val d’Agri oilfield (Italy)
Source: Sci Rep. 2016 Nov 14;6:37165. doi: 10.1038/srep37165 (PMC5107892; doi:10.1038/srep37165)
Supplement: Supplementary Information [file srep37165-s1.doc]

supplementary information for

Inversion of inherited thrusts by wastewater injection induced seismicity at the Val d’Agri oilfield (Italy)

Buttinelli M. ^1^, Improta L. ^1^, Bagh S. ^1^, Chiarabba C. ^2^

*^1^ Istituto Nazionale di Geofisica e Vulcanologia, Department of Seismology and Tectonophysics, Rome, Italy, Via di Vigna Murata 605, 00143, Roma, Italy (*[*mauro.buttinelli@ingv.it*](mailto:mauro.buttinelli@ingv.it)*;* [*luigi.improta@ingv.it*](mailto:luigi.improta@ingv.it)*;* [*bagh.samer@ingv.it*](mailto:bagh.samer@ingv.it)*)*

*^2^ Istituto Nazionale di Geofisica e Vulcanologia, National Earthquake Centre, Rome, Italy (*[*claudio.chiarabba@ingv.it*](mailto:claudio.chiarabba@ingv.it)*)*

Corresponding author: Mauro Buttinelli, Istituto Nazionale di Geofisica e Vulcanologia, Via di Vigna Murata 605, 00143, Roma, Italy ([mauro.buttinelli@ingv.it](mailto:mauro.buttinelli@ingv.it)

**Contents of this file**

Text S1

Figures S1

Caption for Figures S1

Figures S2

Caption for Figures S2

Text S2

Figures S3

Caption for Figures S3

Figures S4

Caption for Figures S4

Table S1

APPENDIX

**Introduction**

The availability of a reliable subsurface model is a basic requirement to comprehend relationships between geological structures and spatiotemporal distribution of natural or induced seismicity. Otherwise, the reliable association of faults imaged by seismic exploration data to earthquake distributions relies heavily on the use of very accurate 3-D absolute locations and source mechanisms, particularly when the velocity structure is extremely heterogeneous, as that of the Val d'Agri (VDA) basin in the southern Apennines of Italy. To address these issues, we analyzed a vast amount of underground dataset to investigate the geological structure below the Costa Molina 2 (CM2) wastewater injector in the VDA. The retrieved faults were compared with accurate 3-D locations and reliable focal mechanisms of the injection-linked seismicity occurred since the beginning of injection (June 2006) nearby the disposal well.

This supplementary information contains details on subsurface data, underground structure, earthquake location and focal mechanisms of induced events located close to the CM2 well.

**Text S1**

We first integrated and completely revised earthquake data recorded by local stations of ENI Oil Company and of INGV in the whole VDA from 2001 to 2014. We analyzed 1200 earthquakes (ML<3.3) recorded at seismic stations belonging to 3 different networks: a dense temporary local network of 27 stations installed by INGV in 2005–2006, the permanent national network of INGV, and the trigger-mode monitoring local network of 15 stations managed by ENI. P- and S-arrival times and P polarities were accurately hand-picked, exploiting, as well, cluster and coherence analysis performed on common station gathers.

After computing preliminary 1-D locations, a total of 1044 local earthquakes were selected based on the following criteria: a root-mean-square (RMS) residual less than 0.5 s, hypocentral errors less than 2.0 km, azimuthal gap less than 180°, and at least 12 P- wave readings. Selected events were recorded by up to 42 local stations installed in the VDA and surrounding regions and were used as input to a local earthquake tomography parameterized by a 3x3x2 km grid node spacing, which is characterized by an unprecedented spatial resolution and hypocenter location accuracy for the VDA oilfield (Bagh et al., 2015).

The 3-D locations of 248 events occurred close to the CM2 well are reported in Figure S1. The seismic events have horizontal and vertical location standard errors lower than 150 and 200 m, respectively. These events cluster between 2.5 and 5 km of depth (b.s.l.) and elongate in the NNE-SSW direction. Events distribution defines a structure dipping ~50° to the NE, with a down-dip extension of about 4 km and a width of about 2.5 km.

The focal mechanisms of events occurred close to the CM2 show predominant normal faulting kinematics in agreement with the local extensional regime that trends SW-NE. Thirty-eight out of 46 M_L_ ≥ 1 events have normal faulting focal mechanism with a minor strike-slip component and nodal planes striking from WNW-ESE to NW-SE, whereas the remaining 8 events show strike-slip high-angle kinematics (Figure S1). Most of the normal-faulting solutions, including the strongest M_L_ 2.2 event, have one of the auxiliary planes dipping 50°-60° toward NE, in good agreement with the fault depicted by alignment of hypocenters.


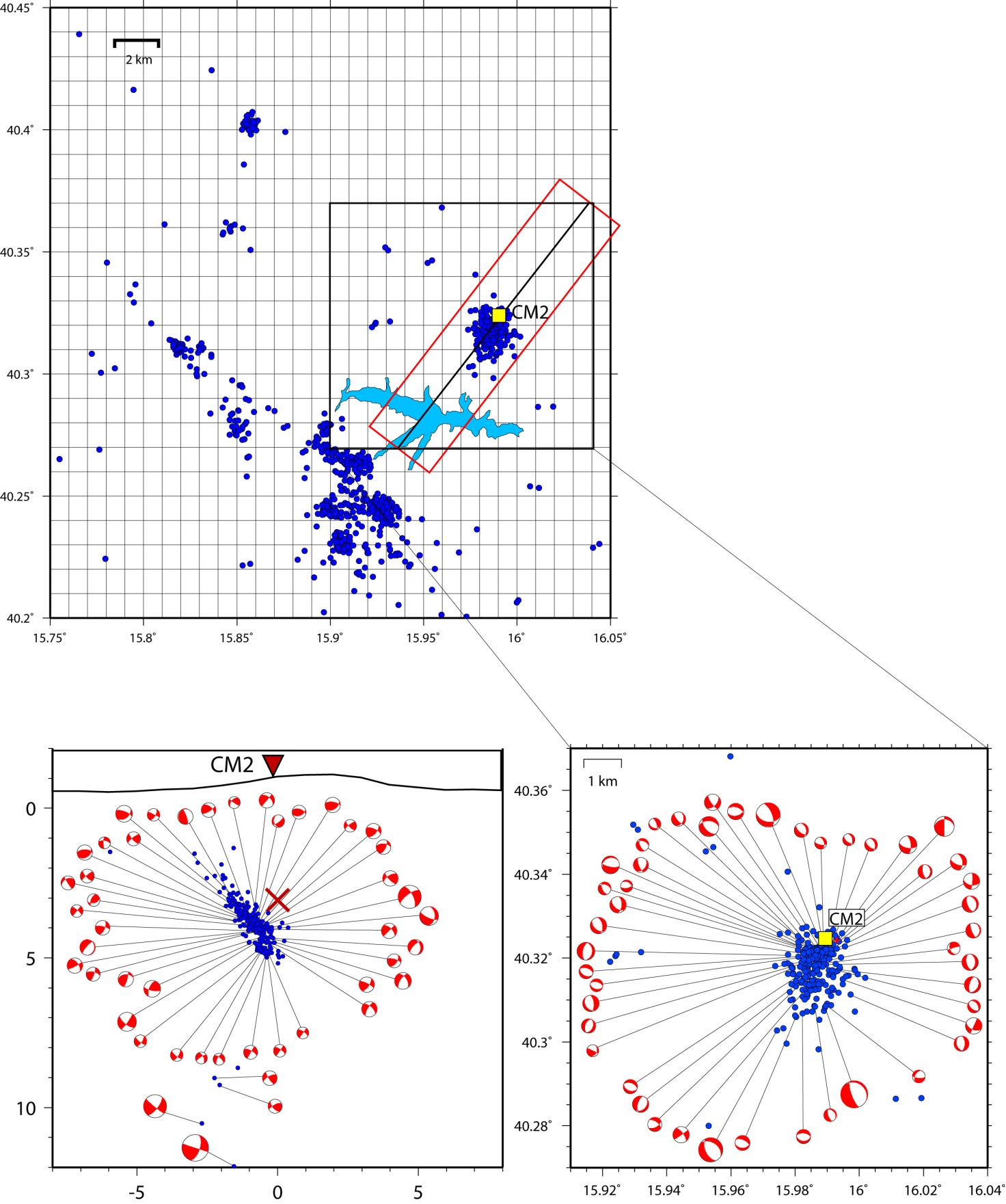


Figure S1. Cross section and map view of the computed focal mechanisms for 46 ML ≥ 1 events occurred close to the CM2 well. The red triangle and cross in the section outline the head and bottom of the CM2 well.

**Text S2**

We analyzed a large amount of hydrocarbon exploration data collected by ENI company in the VDA region and consisting of: i) time-migrated reflection data of a 3-D survey, ii) numerous stack and time-migrated reflection profiles, iii) geophysical and stratigraphic logs from 20 deep wells reaching the API. We also used a few seismic reflection profiles and wells extracted from the ViDEPI database (http:// [www.videpi.com](http://www.videpi.com/), ViDEPI, 2010).

In Figure S2, we provide the location of all the underground dataset used to develop the top API structural map of the injection area. The color-shaded model of the top API surface is reported as a reference, while the depth of the API surface and the location of the exploration wells are already reported in the manuscript (Figure 2B). The depth conversion has been performed referring to the velocity model summarized in Table S1. This model has been computed gathering the velocity information coming from well sonic logs, VSP, seismic checkshots, literature data.

We also provide additional information on the structural style and major tectonic structures of the thrust-and-fold system by including an interpreted seismic profile that runs WSW-ENE from the VDA basin to the eastern sector of the belt (Figure S3, section trace C of Figure 1A).

Our interpretation is constrained by stratigraphic data from wells Costa Molina 2 (CM2) and Tempa del Vento 1 (TV1), surface geological data and take into account underground models available from literature.

The general architecture of this area is dominated by upheaved long-wave anticline deforming the deep Inner Apulia Platform (API), which is un-harmonically covered by a huge tectonic stack of allochthonous units that progressively thickens eastwards. Above the API, the mèlange zone (MEL) consists of a several hundred meters thick succession of deeply deformed Late Miocene-Lower Pliocene mudstones and siltstones including huge exotic blocks of the Lagonegro Basin (LB) imbricates. The MEL seismostratigraphic unit is characterized by a quite chaotic seismic response, while its top corresponding to the overthrust of LB Mesozoic units can be followed with some confidence in correspondence of the API culminations where seismic data interpretation is calibrated by wells. Conversely, in the external, eastern sectors of the belt, it is quite difficult to discriminate the mèlange layer from the overlying thrust sheets because they mainly consist of Tertiary successions of the External Flysch Complex (EFC). The stacking of LB basinal units is more pronounced toward the eastern sector. The eastward thickening of the thrust sheets also corresponds to an abrupt deepening of the underlying API. As reported by other studies, it is quite evident that the thrusts systems affecting the LB units stack have no prolongation into the underneath API structures. Those thrusts end up in correspondence of the regional detachment at the base of the MEL following the thin-skinned deformational style of the “rootless nappes”. Conversely, the thrust systems intersecting API include both blind thrusts that end up into the ductile MEL and major reverse faults that propagated upwards cutting the whole stack of allochthonous units of the accretionary wedge. Those are generally characterized by deeply rooted and steeper thrusts (and associated back thrusts) with respect to those intersecting the LB. Those major thrust sheets are generally associated to large-wavelength anticlines developed in the fault hanging wall and have been related a late Pliocene-Early Pleistocene shortening phase. Moreover, a few structures intersecting the shallow portion of the crust seem to affect at least the LB stack, the EFC units and the allocthnous and wedge top basins deposits, having also a surface expression (e.g., the *Armento thrust fault*). The rooting of such thrust fault express its end into the MEL layer, as already highlighted by several works.

Supplementary information regarding the interpretation of Figure 3 of the manuscript are needed in order to better frame the induced seismicity occurrence in the light of observed geological features. The uninterpreted version of Figure 2A of the manuscript has been provided here in the supplementary material (Figure S4) to provide additional information regarding the geological structure drilled by the CM2 injection well.

Figure 3 illustrates a time migrated seismic section extracted from the 3D seismic dataset, that cross with a WNW-ESE direction the injection area (section trace D of Figure 1A and 2B in the manuscript). The data shows a NNE-SSW trending sub-vertical fault, that matches a rapid southeastward deepening of the Apulian carbonates (Figure 2B). This transverse structure cuts only the Apulian units and the mèlange layer, dips to the SE and is characterized by a minor dextral strike-slip movement. Its origin could be related to a different amount of deformation accommodated by the north-western (larger) and south-eastern (lower) thrust systems during the Late Pliocene-Early Pleistocene shortening. The geometry of this structure matches the NNE-SSW elongation of the seismicity occurring close to the injection well. Thus we hypothesize that this transverse structure represents a barrier for pore-pressure diffusion, partially confining the migration of seismicity, following the mechanisms proposed by Goebel et al., 2016.


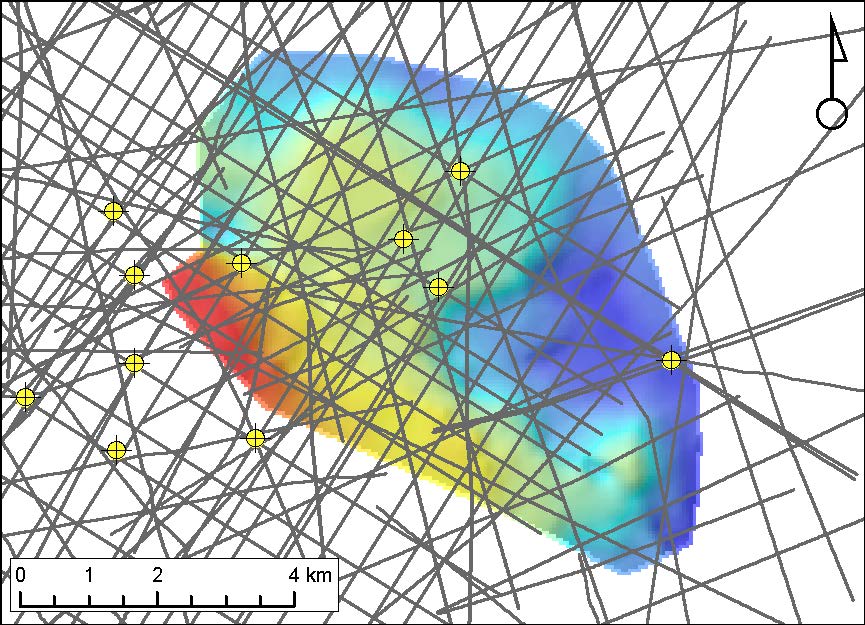


**Figure S2** - Location of seismic 2D reflection profiles, 3-D survey vertical sections and exploration wells used to construct the 3-D structural model and the top API structural map.


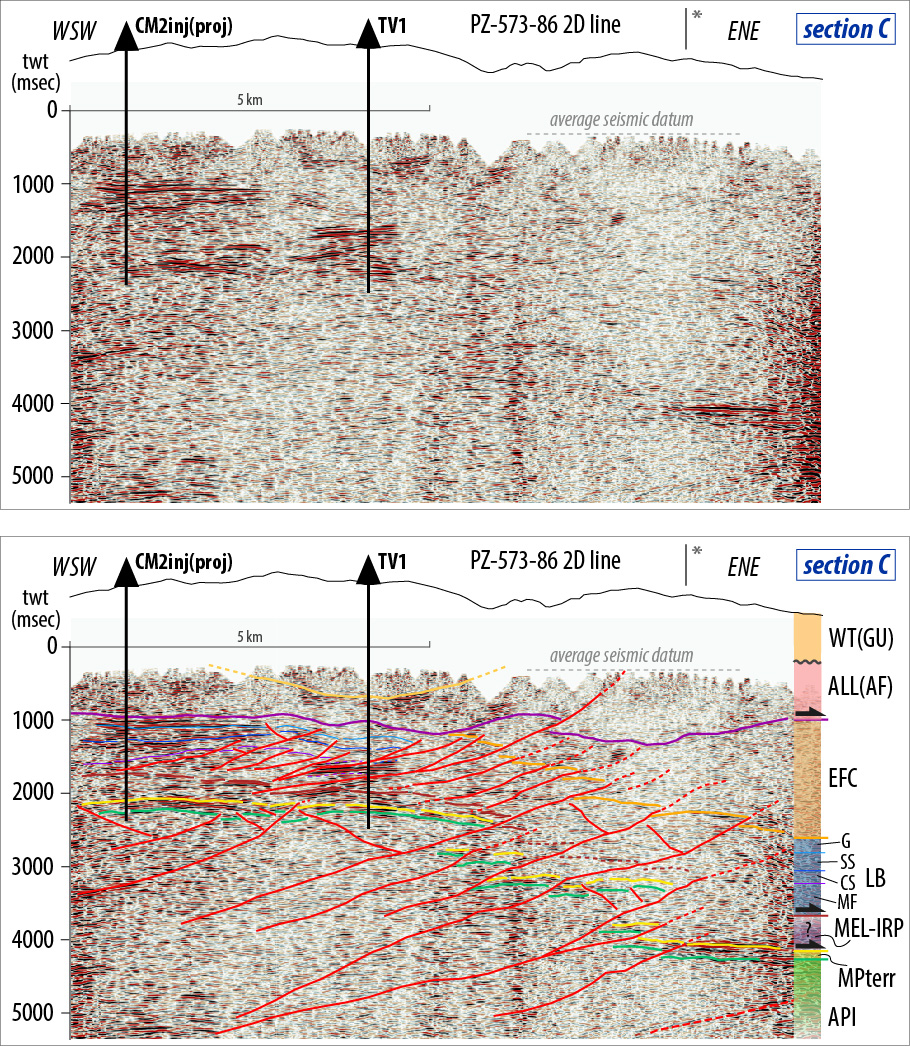


**Figure S3** – Un-interpreted and interpreted versions of a 2D time-migrated seismic reflection profile crossing the injection area (PZ-573-86 line from the ViDEPI public dataset). Section trace in Figures 1A and 2B (section C). Main seismographic units codes references in Figure 1. CM2 well is projected 2 km to the NNW, while the line is tied to Tempa del Vento 1 (TV1) well. The I* marks the end of the trace as reported in Figure 1A.


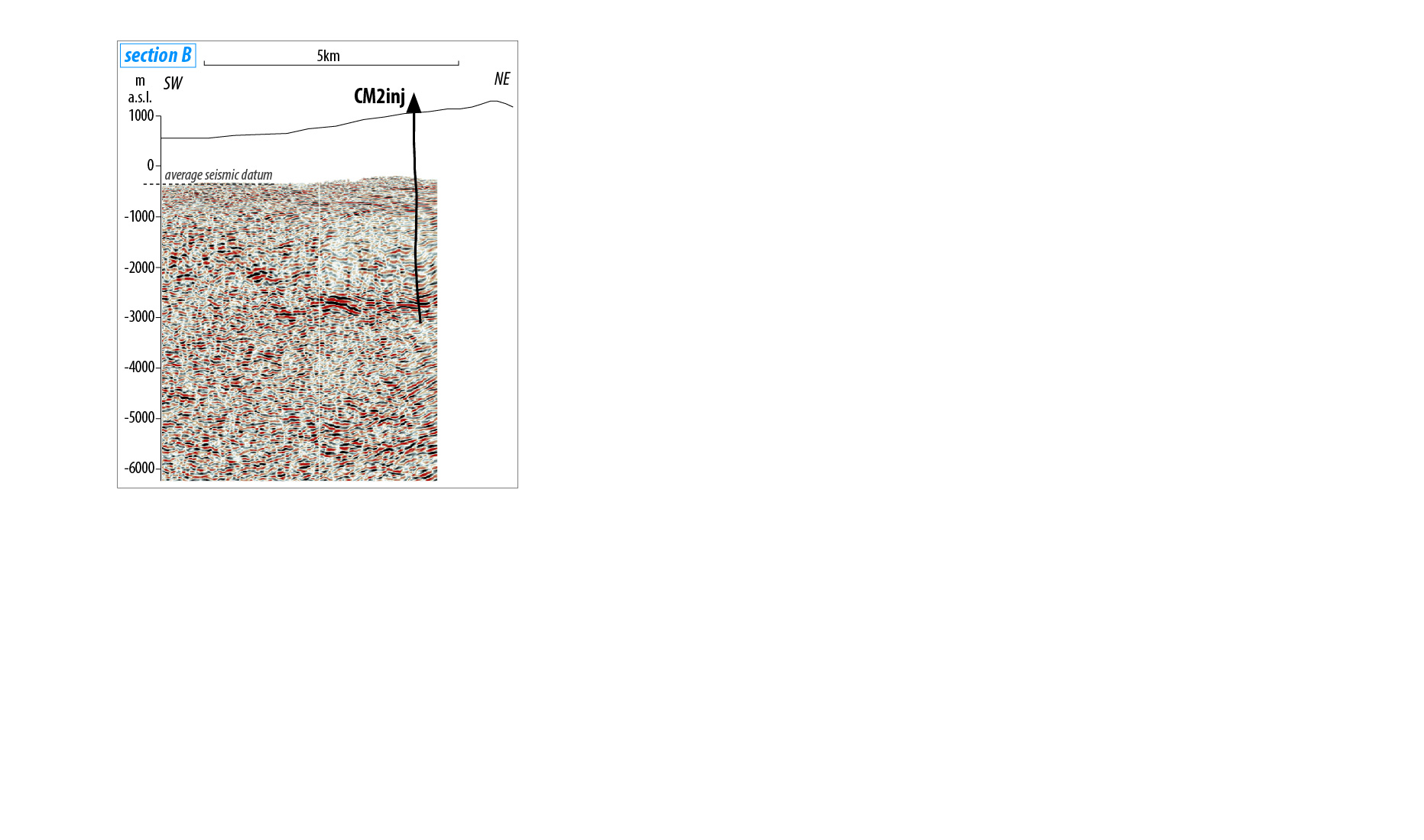


**Figure S4** – Un-interpreted version of Figure 2A of the manuscript. Section trace in Figures 1A and 2B (section B). CM2 well crosses the profile without any projection.


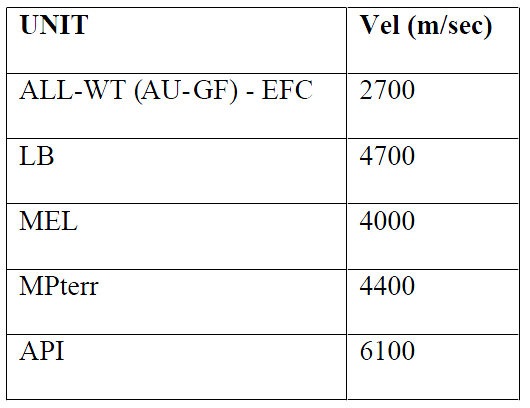


**Table S1** – 1-D velocity model used to perform the depth-conversion of time-migrated data.
